# Full‐field swept‐source optical coherence tomography and neural tissue classification for deep brain imaging

**DOI:** 10.1002/jbio.201960083

**Published:** 2019-12-02

**Authors:** Ilan Felts Almog, Fu‐Der Chen, Suhan Senova, Anton Fomenko, Elise Gondard, Wesley D. Sacher, Andres M. Lozano, Joyce K. S. Poon

**Affiliations:** ^1^ Edward S. Rogers Sr. Department of Electrical and Computer Engineering University of Toronto Toronto Ontario Canada; ^2^ Krembil Research Institute Toronto Western Hospital Toronto Ontario Canada; ^3^ Department of Neurosurgery Centre Hospitalier Universitaire Henri‐Mondor, APHP Créteil France; ^4^ INSERM Unit 955, Institut Mondor de Recherche Biomédicale, Université Paris‐Est Créteil France; ^5^ Max Planck Institute of Microstructure Physics Halle Germany; ^6^ Division of Neurosurgery, Department of Surgery Toronto Western Hospital Toronto Ontario Canada

**Keywords:** image processing, machine learning, optical coherence tomography, optical devices, surgical instruments

## Abstract

Optical coherence tomography can differentiate brain regions with intrinsic contrast and at a micron scale resolution. Such a device can be particularly useful as a real‐time neurosurgical guidance tool. We present, to our knowledge, the first full‐field swept‐source optical coherence tomography system operating near a wavelength of 1310 nm. The proof‐of‐concept system was integrated with an endoscopic probe tip, which is compatible with deep brain stimulation keyhole neurosurgery. Neuroimaging experiments were performed on ex vivo brain tissues and in vivo in rat brains. Using classification algorithms involving texture features and optical attenuation, images were successfully classified into three brain tissue types.
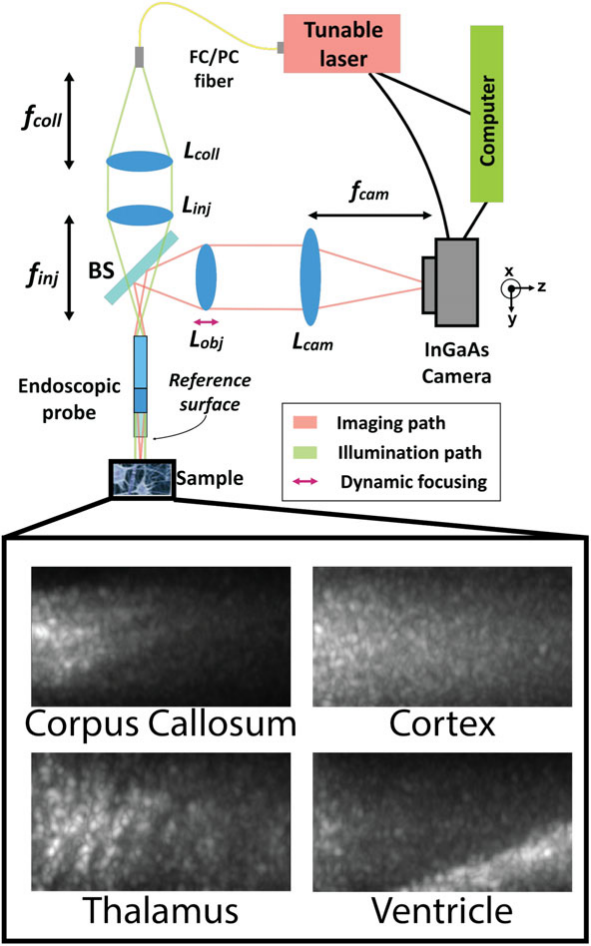

## Graphical abstract

Optical coherence tomography can differentiate brain regions with intrinsic contrast and at a micron scale resolution. We present, to our knowledge, the first full‐field swept‐source optical coherence tomography system operating near a wavelength of 1310 nm. The proof‐of‐concept system was integrated with an endoscopic probe tip, which is compatible with deep brain stimulation keyhole neurosurgery. Using classification algorithms involving texture features and optical attenuation, images were successfully classified into three brain tissue types.

## 1 INTRODUCTION

Neurosurgical interventions have evolved to provide effective treatment for a variety of disabling conditions including tumors 1, epilepsy 2, dystonia 3 and Parkinson's disease 4, 5, 6. Given the small scale and intricate complexity of deep brain structures, accurate localization of structures and guidance of instruments have a significant impact on clinical outcomes. However, current intraoperative guidance methods are limited by an absence of real‐time visual feedback of the region ahead of surgical instruments inserted in the brain. Although auxiliary imaging technologies have been developed such as intraoperative magnetic resonance (MR)/computed tomography (MR/CT)‐assisted neuronavigation 7, 8), they fail to provide real‐time feedback of the precise position of specific regions and structures targeted by the neurosurgical procedure 9, 10. A common minimally invasive technique in stereotactic neurosurgery is the implantation of electrodes for deep brain stimulation (DBS) 5, 11. The current standard for guidance in DBS electrode implantation surgery is the recording of electrical neuronal activity via microelectrodes 12. Different brain regions are identified by the electrophysiological pattern of neuronal activity, or by the patient's behavioral response to electrical stimulation at the implanted electrode 13, 14, 15. Such iterative recordings and stimulation are lengthy procedures and require an expertise in electrophysiology which might not be available in all neurosurgical centers.

Optical coherence tomography (OCT) is an imaging technique which can generate high‐resolution 3D images, in some cases in real time. OCT first became popular in the field of ophthalmology, allowing for improved diagnosis of retinal conditions. OCT is now the gold standard in retinal imaging and its use in many other clinical fields has been investigated 16, 17, 18, 19, 20, 21, 22, 23, 24, 25. More recently, OCT has been used to differentiate brain elements with intrinsic contrast (ie, without additional labeling) and appears to be a promising option for neurosurgical guidance 26, 27, 28, 29, 30, 31, 32.

Swept‐source OCT (SS‐OCT), a type of OCT which employs tunable laser sources, has been shown to provide increased sensitivity and speed 33. Although SS‐OCT has been previously implemented with full‐field (FF) acquisition 34, 35, 36, in which 2D frames are acquired with a camera instead of point‐by‐point scanning, no such systems have been shown employing wavelengths around 1310 nm. As that is a standard wavelength band in telecommunications, laser sources, optical components and specialized coatings are widely available. The wavelength also coincides with one of the biological optical windows, providing longer imaging depth in tissue 37, 38, 39, 40. Such specification is critical in detecting upcoming vascular structures for surgical guidance applications. Moreover, to our knowledge, no FF‐SS‐OCT system has been demonstrated with an endoscopic tip, which is small enough to fit into the surgical cannula to access deep brain regions.

Several studies have evaluated the feasibility of using OCT for the visualization of morphological structures in the brain and to distinguish between healthy and diseased (eg, tumorous) brain tissue. These systems relied on indirect features other than intrinsic brain structures, such as signal attenuation or the presence of blood vessels, calcifications or cysts, to achieve classification of tissue 41, 42, 43, 44. More recent studies overcame these limitations and were able to resolve fine morphological features of the brain 27, 28, 30, 45. However, all of these studies used large microscope objectives which can only image tissue sections or the surface of the brain and are incompatible with deep brain imaging.

An endoscopic FF‐OCT system demonstrated by Benoit a la Guillaume et al provided sufficient resolution to resolve neurons, but its imaging depth was limited to tens of microns 46, whereas the structures of interest for neurosurgeical procedures are at least 1 mm thick. Another study, by Liang et al, employed an endoscopic probe in a SS‐OCT system around 1310 nm and was able to distinguish between white matter, gray matter and blood vessels in a sheep brain, which has a brain volume that is about 100 times larger than a rat, but the authors could not distinguish not between among different types of gray matter 32. Also, although the endoscopic probe diameter was thinner than ours (740 μm vs 1 mm), that system used a scanning mechanism and only produced 2D images. In the context of DBS, acquiring a 3D volume of several hundred microns can be a valuable supplement in precisely identifying topologically complex brain structures.

In this work, we present the first FF‐SS‐OCT with an endoscopic probe tip operating in the 1310 nm wavelength range. We designed the system to be compatible with neurosurgical requirements. Images were obtained from both ex vivo brain tissue and in vivo animals. Image processing algorithms were developed and applied to the obtained images for the differentiation of brain regions.

## 2 MATERIALS AND METHODS

### 2.1 Optical design

**Figure 1:**
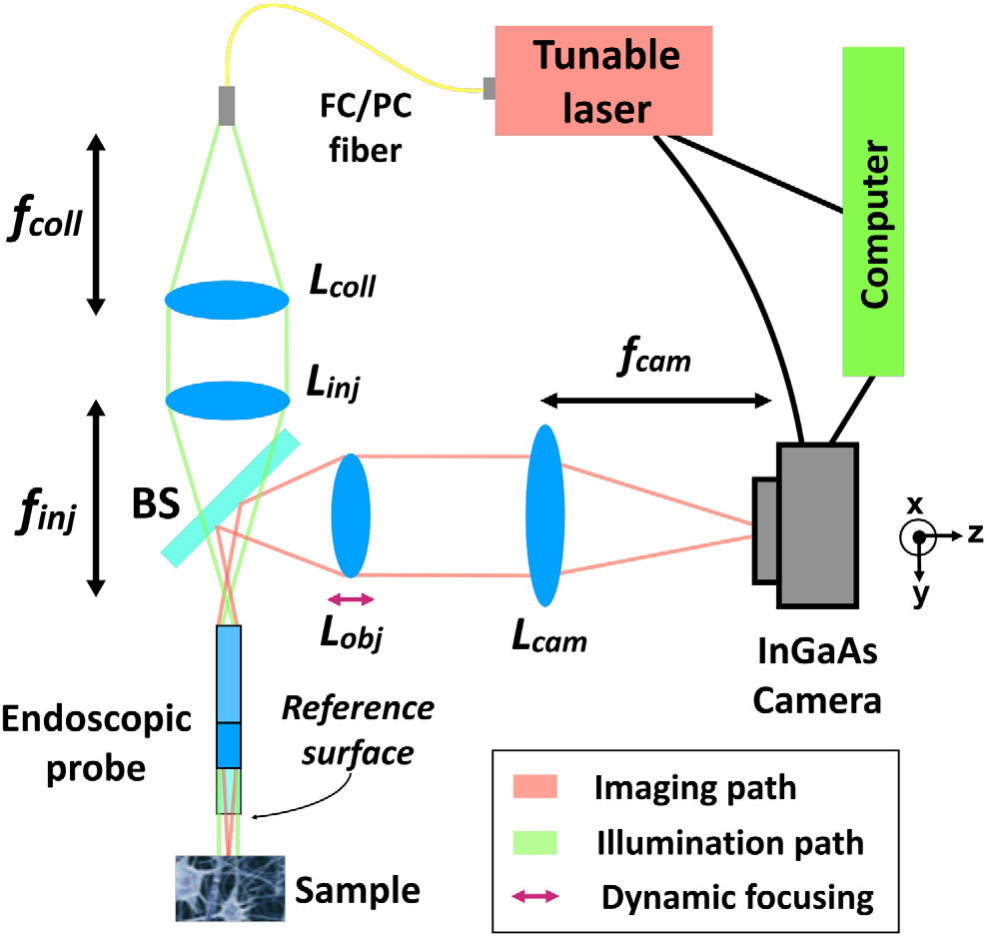
A diagram of the entire FF‐SS‐OCT system. *L*_coll_: collimation lens with focal length *f*_coll_ = 75 mm. *L*_inj_: injection lens with focal length *f*_inj_ = 75 mm. *L*_obj_: objective lens of *f*_obj_ = 75 mm, which can be translated for dynamic focusing. *L*_cam_: camera lens with focal length *f*_cam_ = 1000 mm. BS, beamsplitter

Figure 1 shows the overall design of the OCT system. The endoscopic probe was the most essential component in the system. The probe design was carried out via Zemax OpticStudio simulations and its components were custom‐manufactured. A low numerical aperture (0.2 NA) cylindrical gradient‐index (GRIN) rod lens (GRINTech GT‐LFRL‐100‐20‐CC‐1550) of 1 mm diameter and cut to 3.18 mm length was attached to a cylindrical glass spacer (7.1 mm long). This arrangement provided for a longer depth of field (DOF) while meeting the desired resolution and magnification requirements for object planes within ~150 μm away from the endoscope facet. The total length of the endoscope was 10.28 mm, which could be increased for deeper penetration into the brain with the addition of a relay GRIN rod lens. The diameter of the endoscope increased to 1.5 mm after the addition of a stainless steel sleeve, which fits in most neurosurgical cannulas 47. We used the reflection from the distal surface of the endoscope as the reference for the common‐path interferometer.

Achromatic doublets and a beamsplitter (BS) were used for relaying light into the endoscope and for relaying the interference image to the infrared camera (Xenics Xeva 320‐1.7) via two 4f arrangements, as shown in Figure 1. These 4f arrangements allowed for controlling the diameter of the collimated beam entering the endoscope, optimizing power coupling, as well as to choose the total magnification of the imaging system. Light was injected into the system with a standard FC/PC SMF28 fiber. A summary of the achieved parameters for the setup specification is listed in Table 1.

**Table 1:** Summary of measured metrics for the complete optical system

Parameter	Measured value
Endoscope length	10.28 mm
Endoscope diameter	1.5 mm (with sleeve)
Total magnification	×21.17
NA	0.102
*f*#	8.35
Transverse resolution	8.77 μm (in air)
FOV	450 μm × 450 μm
Axial resolution	14 μm (in agarose gel)
Depth of field	400 μm
Working distance	variable
Optical loss	−5 dB
Sensitivity	47 dB
Acquisition + processing time	<20 seconds

### 2.2 OCT‐resolving steps

Identical to other SS‐OCT systems, the interference pattern on the camera, *I*_IMG_, of the proposed FF‐OCT system can be given as

(1) $$\begin{array}{r} I_{\mathrm{IMG}}\left(x_{0}^{'},y_{0}^{'}\right)\propto I_{\mathrm{ref}}+\sum_{i}^{\max}\left\{I_{samp}\left\{x_{0},y_{0},z_{i}\right\}+2\sqrt{I_{\mathrm{ref}}I_{\text{samp}}\left\{x_{0},y_{0},z_{i}\right\}}\cos\left[2\left[\frac{2\pi n}{\lambda_{0}}\right]\left[z_{i}-z_{\mathrm{ref}}\right]\right]\right\}\\ +\sum_{m,n\cap m\neq n}^{\max}2\sqrt{I_{\text{samp}}\left(x_{0},y_{0},z_{m}\right)I_{\text{samp}}\left(x_{0},y_{0},z_{n}\right)}\cos\left[2\left[\frac{2\mathrm{\pi n}}{\lambda_{0}}\right]\left[z_{m}-z_{n}\right]\right] \end{array}$$

where *z*_ref_ is the depth of the reference, *z*_max_ is the maximum distance from which backscattered light is observable, *n* is the refractive index of the sample, *I*_samp_(*x*_0_, *y*_0_, *z*_*i*_) is the intensity of backscattered light from each reflector at location (*x*_0_, *y*_0_, *z*_*i*_) in the sample and *I*_ref_ is the intensity of backscattered light from the reference reflector.

In FF‐SS‐OCT, by sweeping wavelength *λ*_0_ and applying the Fourier transform on the interference pattern *I*_IMG_, depth information can be retrieved 48. In the full field configuration, the detection was done on the entire 2D field rather than a single point source, which shortens data acquisition time and removes the need for a scanning mechanism.

Our system used a swept wavelength tunable laser from 1260 to 1345 nm (Keysight 81600B‐130). The wavelength step size was 0.042 nm to yield over 2020 frames per sweep, translating to 10 mm in depth. The step size was chosen such that any aliasing noise from the interface or proximal end of the GRIN lens would not be within the usable DOF range of the optical system.

Custom code for hardware control image acquisition and processing was implemented in Matlab. A volumetric sweep (C‐scans) with a volume of 450 × 450 × 700 μm^3^ can be acquired, processed and displayed in less than 20 seconds on a computer with and 3.60 GHz Intel Core i7 CPU and 16 GB of random access memory. The depth information is extended beyond the DOF because some structures are still preserved in deeper region. Such image has been included in the Supplementary Document S2.

**Figure 2:**
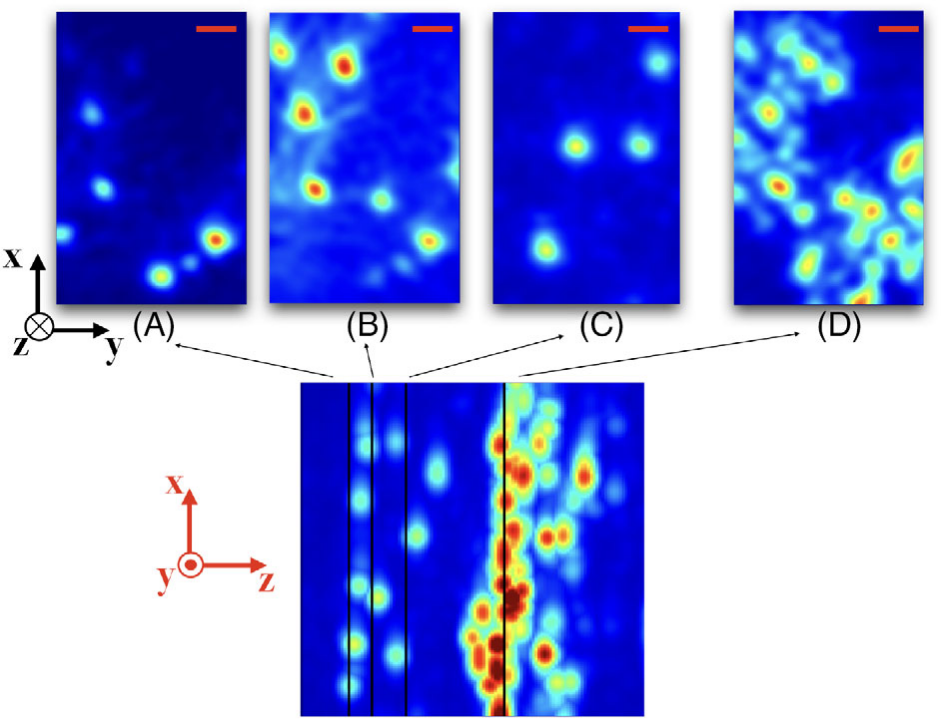
Resolved images from a phantom made of 55 μm glass beads in agarose gel at depths of (A) 40 μm, (B) 50 μm, (C) 90 μm and (D) 177 μm from the reference surface. Scale bar (red): 40 μm. The bottom image shows a maximum intensity projection along the *y* dimension (spanning a total of 305 μm along *z*) and the positions from where the slices above were taken. The thick layer of beads was a result of deposition at the bottom of the phantom during the gelation of the agarose. Axes are shown in black for the slices and in red for the projection depth map

As a validation of the proposed OCT design, we resolved a volumetric image of a phantom made of glass beads with a diameter of 55 ± 1 μm and a refractive index of 1.95 in 1% agarose gel (Corpuscular Corbeads 4‐55). The results presented in Figure 2 demonstrated the system can resolve beads at various depths.

### 2.3 Experimental procedures

All animal procedures conformed to ethical guidelines as per the Canadian Council for Animal Care. Two adult Wistar rats (200‐300 g) were used for the in vivo experiments and three rats were used for the ex vivo experiments.

For ex vivo experiments, the animal was humanely euthanized with urethane overdose, decapitated, and the entire brain was then extracted and immediately frozen, then sliced and thawed at room temperature. Ex vivo images were acquired within 30 minutes of thawing from the cortex, the corpus callosum, the striatum, the hippocampus, the thalamus and the ventricle by sectioning the brain and directly placing the endoscope on the targeted region. However, thawed brain samples were relatively deformable. To validate whether the signal is repeatable over sequential scans at the same position, some brain samples were maintained in a frozen state during data acquisition.

For in vivo experiments, the rats were anesthetized with an intraperitoneal injection of 1.5 g/kg of urethane and mounted with earbars on a stereotactic frame (Kopf Instruments). The stereotactic frame was vertically mounted to a custom rigid frame holder on an *xyz*‐translation stage. Bilateral cranial opening over the frontoparietal region was performed, and the dura mater was punctured at the cortical penetration location (Bregma: ML −3 mm, AP −2.3 mm) prior to inserting the endoscope, the surface of the brain was irrigated and the dura mater at the planned penetration location was punctured prior to inserting the endoscope.

The system was aligned with the aid of the USAF‐1951 resolution target. Camera settings were adjusted for fast acquisition of images with high dynamic range. The integration time was adjusted to maximize the acquired intensity without saturation over all wavelengths and all pixels. The target region of the brain was positioned in front of the tip of the endoscope and aligned with the aid of the translation stages. The animal was then moved towards the endoscope until a fluid interface covered the endoscope facet. For in vivo experiments, this was the reference position with respect to which penetration depths were measured. The entire stereotactic frame was slowly advanced as the endoscope penetrated into the brain by 500 μm or 2 mm steps, depending on the region.

At least three sequential sweeps were acquired at each position. Upon completion of the experiments, the endoscope was gently retracted, and the tract was irrigated with saline until bleeding was stopped. Transcardiac perfusion with 250 cc of 0.9% saline and 250 cc of 4% paraformaldehyde fixative solution was used to euthanize the animals under deep anesthesia, followed by decapitation. The brains were removed and sectioned into 40 μm thick slices for histological staining. Nissl‐stained sections were then compared to the OCT images to correlate the features with the Rat Stereotactic Brain Atlas 49.

## 3 RESULTS

### 3.1 Ex vivo brain imaging

**Figure 3:**
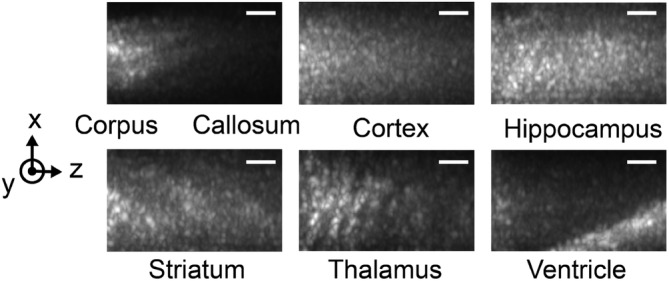
Ex vivo tissue optical coherence tomography (OCT) images of brain regions showing different structural features. The images are maximum projections along the y‐axis to reveal the dominant features in the volumetric image. Scale bar: 50 μm

OCT scans of ex vivo brain tissues, sliced to expose the different regions of interest, were obtained by directly placing the endoscope on the targeted region. Experiments were done on the cortex, the corpus callosum, the striatum, the hippocampus, the thalamus and the ventricle. Figure 3 shows representative images of the brain regions with maximum intensity projection along the y‐axis over 161 frames, equivalent to a depth of 450 μm. A total of 53 volumetric images were collected (to be discussed in the next section), and they are similar to the results shown in Figure 3. The distinct features in the images relate to the optical properties of each region. For instance, due to stronger scattering in white matter, light is more attenuated in the corpus callosum. Cortex and hippocampus have a homogeneous pattern, whereas striatum and thalamus exhibit dark stripes along the axial direction, partly due to a more heterogeneous structures in the region. Lastly, the ventricle appears dark until deeper regions, where tissues scatter light more strongly. The image characteristics are quantitatively analyzed for classification in the next section.

**Figure 4:**
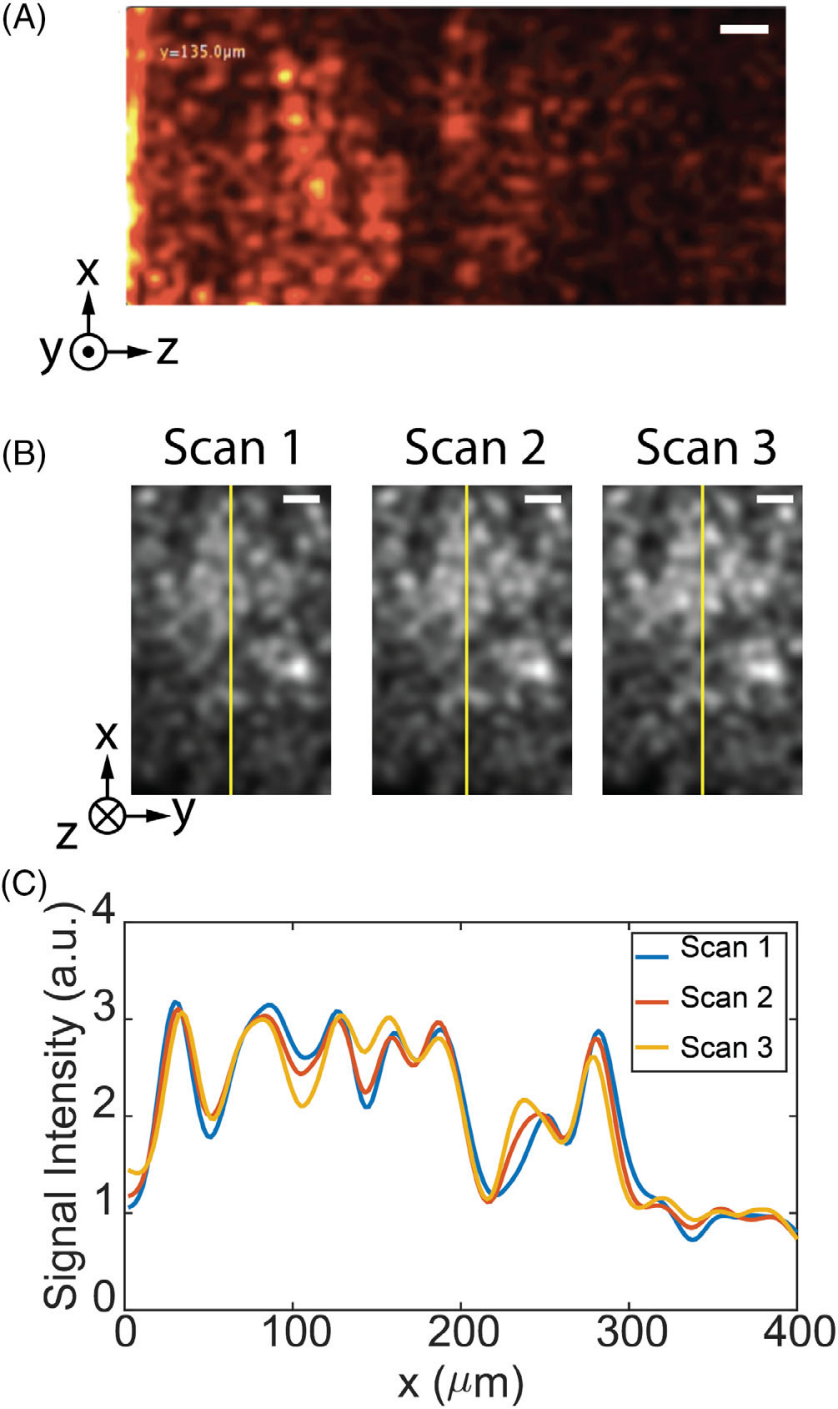
Ex vivo tissue imaging. A, Optical coherence tomography (OCT)‐resolved depth profile (B‐scan) of the thalamus region in room temperature. Scale bar: 30 μm. B, OCT‐resolved cross‐sections at a depth of 450 μm for three successive scans of the frozen tissue in the thalamus. Scale bar: 50 μm. C, A comparison of the intensity profile along the yellow line indicated in (B), confirming the repeatability of the scans

Without maximum intensity projection, it was more challenging to observe the distinct features but some macro structures in certain brain regions could be captured. An example of a B‐scan of an ex vivo rat thalamus is shown in Figure 4A. Macroscopic (>10μm in size) features are observed even several hundred micrometers deep in the tissue, but cellular features, such as neuron bodies, could not be identified. The dark regions in Figure 4A have depths of about 50 microns, which may correspond to blood vessels or high concentrations of cell bodies. Vessels and cell bodies have been reported to appear dark when imaged by OCT 30.

The scans were reproducible if the tissue did not change. Figure 4B shows the cross‐section of frozen thalamus tissue at a depth of 450 μm for three successive scans. Although the smallest features had some variation, the overall images and features are reproduced repeatably with high fidelity. Figure 4C compares the signal intensity along the yellow line in Figure 4B. The intensity profiles of the three successive scans are nearly identical across the field of view. We apply the Matlab Structural Similarity evaluation function (SSIM) to two sequential sweeps to quantify the similarity of images. This metric measures the similarity of two images based on (a) luminance, (b) contrast and (c) structure, and has a value ranging from −1 to 1, with identical images having a value of 1 50. The SSIM value for two sequential OCT reconstructions at a 450 μm depth in the tissue over 10 trials is 0.91 ± 0.03. The error is likely due to thawing process of the samples when in contact with the endoscope.

### 3.2 Classification on ex vivo data

To further understand the significance and the consistency of the features observed in the ex vivo data, we have built a classification model to differentiate the brain regions. To test the repeatability of the features, we collected a total of 53 volumetric images from nonperfused ex vivo samples from three rats. These images were obtained with precise knowledge of the location of the endoscope tip during the scan time, providing a ground‐truth for classification. Each C‐scan was split into two C‐scans along the y‐direction to increase the sample size, resulting in a total of 106 data sets. Further splitting of the data degrades the image consistency within the same group because image intensity falls off on the side of the image a result of Gaussian illumination. Finally, the images were grouped into three classes: Group 1: cortex or hippocampus (44 samples); Group 2: corpus callosum (24 samples) and Group 3: striatum or thalamus (38 samples). These classes were selected due to their similarity in macro‐scale features captured in OCT images as described in previous section. Also, the cortex and hippocampus can belong in the same class because they are located at distinctly different depths of the brain, and thus can be clearly differentiated according to the depth of penetration of the endoscope. Similarly, the insertion location can differentiate regions in Group 3 (striatum/thalamus) since the striatum is located in the anterior of the brain while thalamus is located at the posterior region. The ventricle is not included in the classification subgroup because of limited data. The data set is split into a training set (70 samples) and a test set (36 samples). Training and testing sets are sampled from different rats for validating the generalization of the classifier across multiple animals.

**Figure 5:**
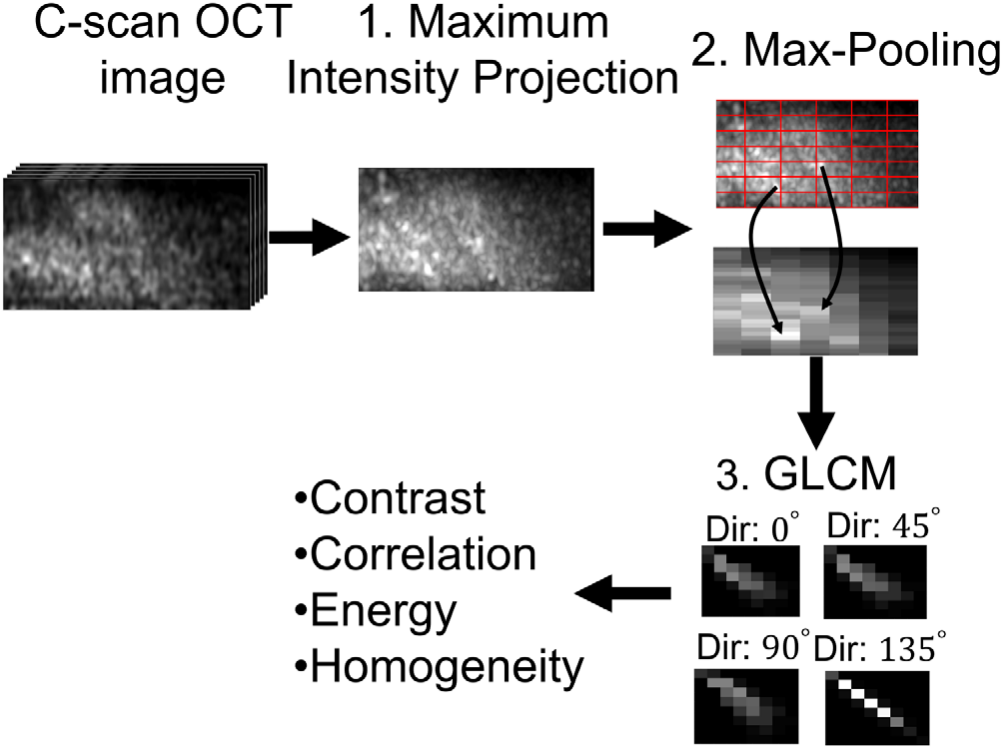
A summary of the flow diagram to extract Gray Level Co‐Occurrence Matrix (GLCM) properties from optical coherence tomography (OCT) images

For feature extraction, we used the Gray Level Co‐Occurrence Matrix (GLCM) for capturing the texture features of the image and attenuation coefficient for describing the optical properties the tissue type. GLCM is a common texture‐based feature which evaluates four properties of a pixel value with respect to its neighbors: (a) contrast, (b) uniformity of energy, (c) correlation and (d) homogeneity, to provide a statistical measure of the intensity variation in space 51. Several processing steps were applied to the image before extracting GLCM properties. A summary of the processing steps is presented in Figure 5 which we describe in the next paragraphs.

**Figure 6:**
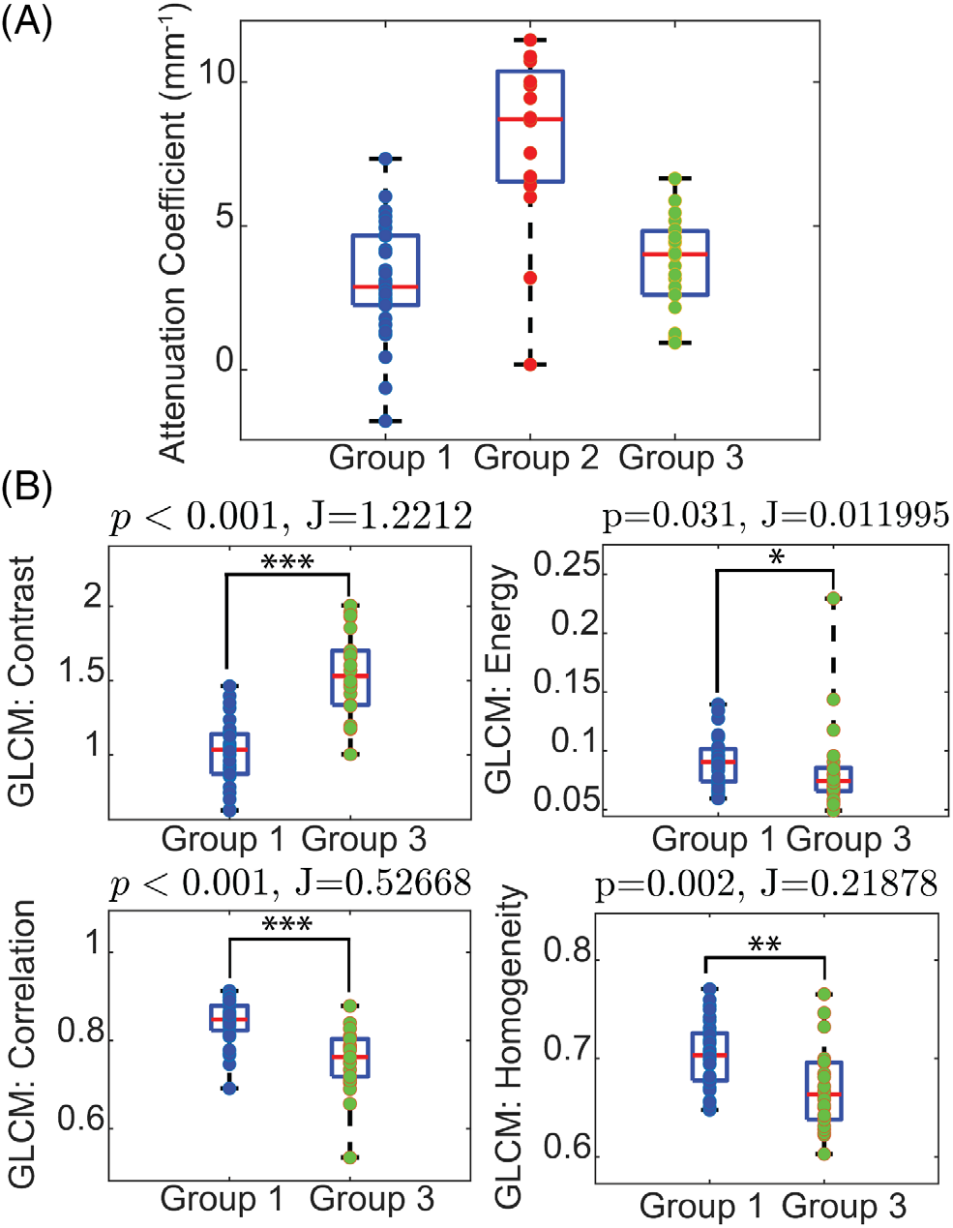
Quantitative analysis of image properties. Group 1: Cortex/Hippocampus, Group 2: Corpus Callosum, Group 3: Striatum/Thalamus (A) The distribution of attenuation extracted from all data sets from the three groups. The corpus callosum has a higher mean attenuation coefficient than gray matter. B, A comparison of the class separability, *J*, of the four Gray Level Co‐Occurrence Matrix (GLCM) properties as well as the statistical significance difference between the two groups validated with Mann‐Whitney *U* test. Contrast and correlation achieve high separability with statistical significance (*P* < .001) between Groups 1 and 3

Previous works 29, 52, 53 have shown that the attenuation coefficient of tissue calculated from the intensity decay along the imaging depth can differentiate between white and gray matters, because white matter, consisting of myelinated neural fibers, scatters light more strongly. Figure 6A shows a similar results in our data, as the attenuation from the corpus callosum (Group 2) is clustered on the higher side of the attenuation axis. However, we can see from Figure 6A that attenuation is insufficient in separating different types of gray matter (Groups 1 and 3).

Instead, we found more success using GLCM to extract features that enabled us to differentiate between homogeneous gray matter and other brain regions with more complex structures. To prepare the image data for GCLM, first, a maximum intensity projection was performed along the y‐axis to collapse the prominent features into a single frame followed by max‐pooling into a grid size of 25 by 5. These two downsampling schemes are essential to capture the sparse features scattered across the image. Then, the four GLCM properties were extracted with a step distance of one pixel along four directions (0°, 45°, 90° and 135°). The mean of the properties across the four directions was used to minimize the effect of rotational variance.

To understand the significance of the features in differentiating the two groups, we calculated the *P*‐value with Mann‐Whitney *U* test. We also used the ratio of between‐ and within‐class scatter matrices to evaluate the separability of the cluster 54. The formulation of the metric can be written as

(2) $$J=\frac{S_{B}}{S_{W}}\;,$$

where *S*_W_ is the within‐class scatter matrix and *S*_B_ is the between‐class scatter matrix.

*S*_W_ is defined as

(3) $$S_{W}=\sum_{i=1}^{C}\sum_{x\in D_{i}}\left(x-\mu_{i}\right){\left(x-\mu_{i}\right)}^{T}\;,$$

and *S*_B_ is defined as

(4) $$S_{B}=\sum_{i=1}^{C}N_{i}\left(\mu_{i}-\mu \right){\left(\mu_{i}-\mu \right)}^{T}\;,$$

where *C* is the number of classes, *x* is the data points in class *i*, *μ*_*i*_ is the mean of *x*, *μ* is the mean of all data points and *N*_*i*_ is the number of samples in each class. This ratio compares the distance of between each group with the variance within each group. A larger ratio indicates the groups are further apart from each other.

Figure 6B shows a comparison of the four GLCM properties between Group 1 and 3. As expected, the image contrast of striatum/thalamus is higher than homogeneous regions like cortex/hippocampus with statistical significance. Higher correlation for images of cortex/hippocampus also suggests that the pattern is more homogeneous. The separability metric, J, and the *P*‐value suggest that contrast and correlation perform relatively well in discriminating cortex/hippocampus from striatum/thalamus; thus, both are features used in the classification.

**Figure 7:**
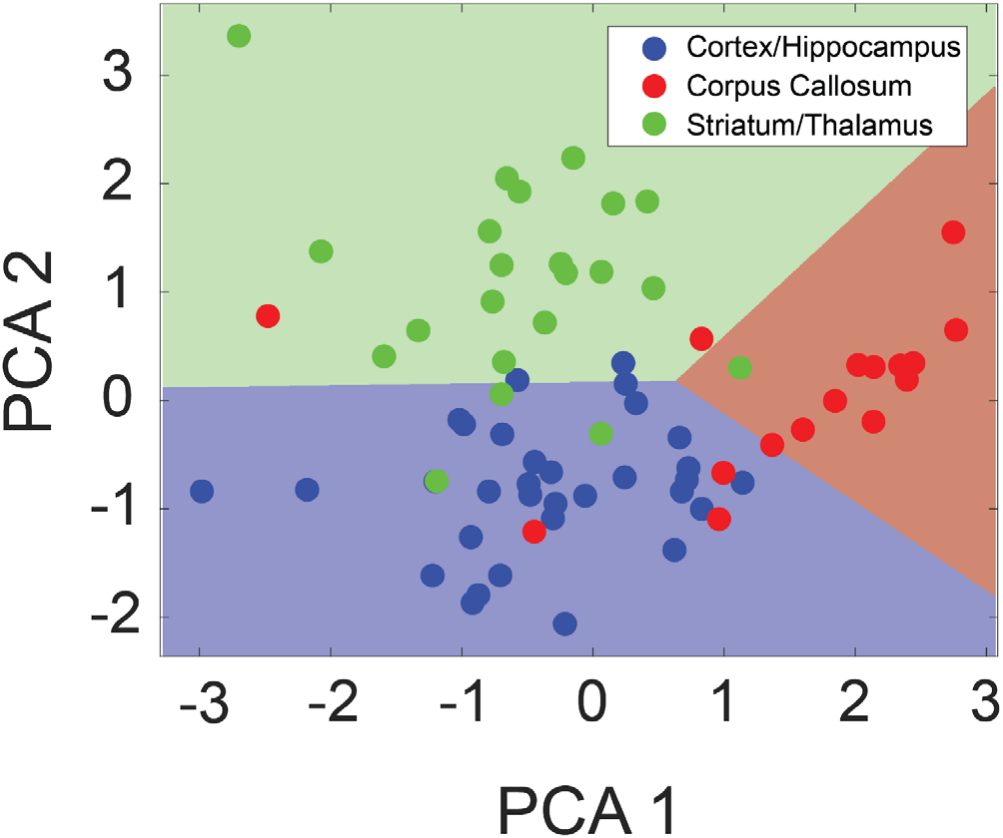
Results from tissue classification based on the attenuation and Gray Level Co‐Occurrence Matrix (GLCM) properties. Dimensionality‐reduced principal component analysis (PCA) plot showing the classification of tissue types into (a) cortex/hippocampus, (b) corpus callosum and (c) striatum/thalamus. The linear hyperplane formed by the support vector machine classifier is overlaid to reveal the classification accuracy. Roughly 80% of the data are labeled correctly with some misclassified data potentially due to tissue variability

Figure 7 shows data plotted in the two dominant components in the principal component analysis and the hyperplane of support vector machine was overlaid on top. There is a clear tendency that the data from each class form a cluster even though there are some data overlapped, partly due to tissue variability like the density or the orientation of myelinated axon. With this method, the OCT images were classified into three tissue groups with 10‐fold cross‐validation accuracy achieving 82.4% ± 14.7% and a test accuracy of 75%, indicating the model is generalizable over the three rats.

### 3.3 In vivo brain imaging

**Figure 8:**
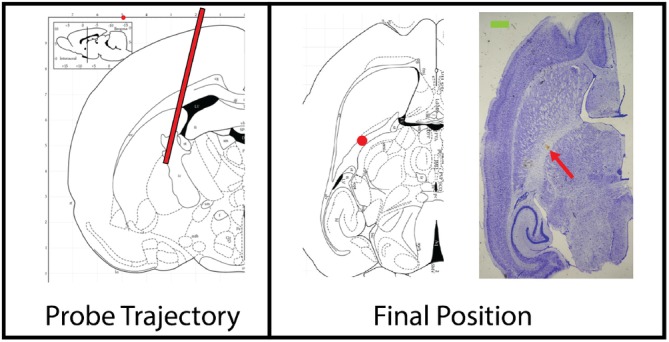
Insertion trajectory of the endoscope for in vivo experiment labeled with a red line. Horizontal section from the Stereotaxic Rat Brain Atlas showing the final position of the endoscope (red dot): Bregma: ML −4 mm, AP −2 mm, DV +5 mm), located within the internal capsule, inferior to the putamen. The blood stain pointed by the red arrow on the Nissl‐stained image on the right confirmed the final position of the endoscope. Scale bar: (green) 1 mm 49

For the in vivo experiments, we targeted the putamen, a part of the striatum which is particularly relevant for functional neurosurgery and the treatment of Parkinson's disease 55. Here, we present an insertion performed on one rat. The penetration tract for the experiment is shown in Figure 8. The path of the tract and the end position were confirmed using Nissl‐stained histology.

**Figure 9:**
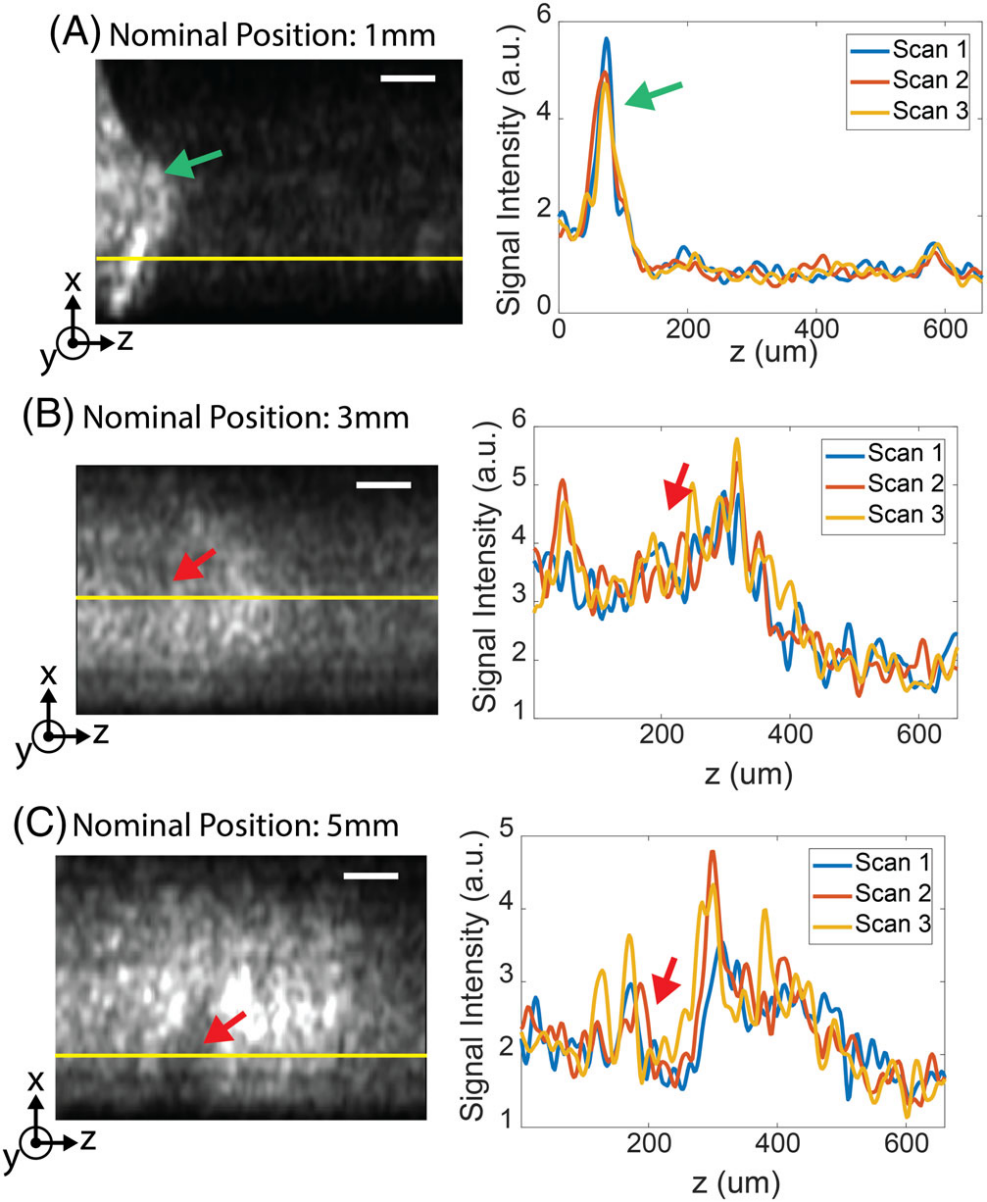
Selected frames showing optical coherence tomography (OCT)‐resolved depth along the tract, nominally at (A) 1 mm, (B) 3 mm and (C) 5 mm into the brain. The line profiles of the three repeated sweeps along the yellow line are presented. Some structural features, indicated by the red arrows, are preserved over time. The green arrow in (A) indicates potential tissue residue. Scale bar: 100 μm

Figure 9 shows the reconstructed images at several penetration depths. The bright structure indicated by the green arrow in Figure 9A was likely tissue residue stuck on the endoscope during the insertion process, since such structure appeared consistently for penetration depths from 0 up to 2 mm. The first 140 μm was cropped for the following analysis for all images to avoid the influence of the tissue residue. In Figure 9B,C, some dark shadows, as indicated by the red arrows, may be blood vessels since blood is more absorbing than other tissue types at 1310 nm. These features appeared consistently over three repeated scans as presented by the line profile.

**Figure 10:**
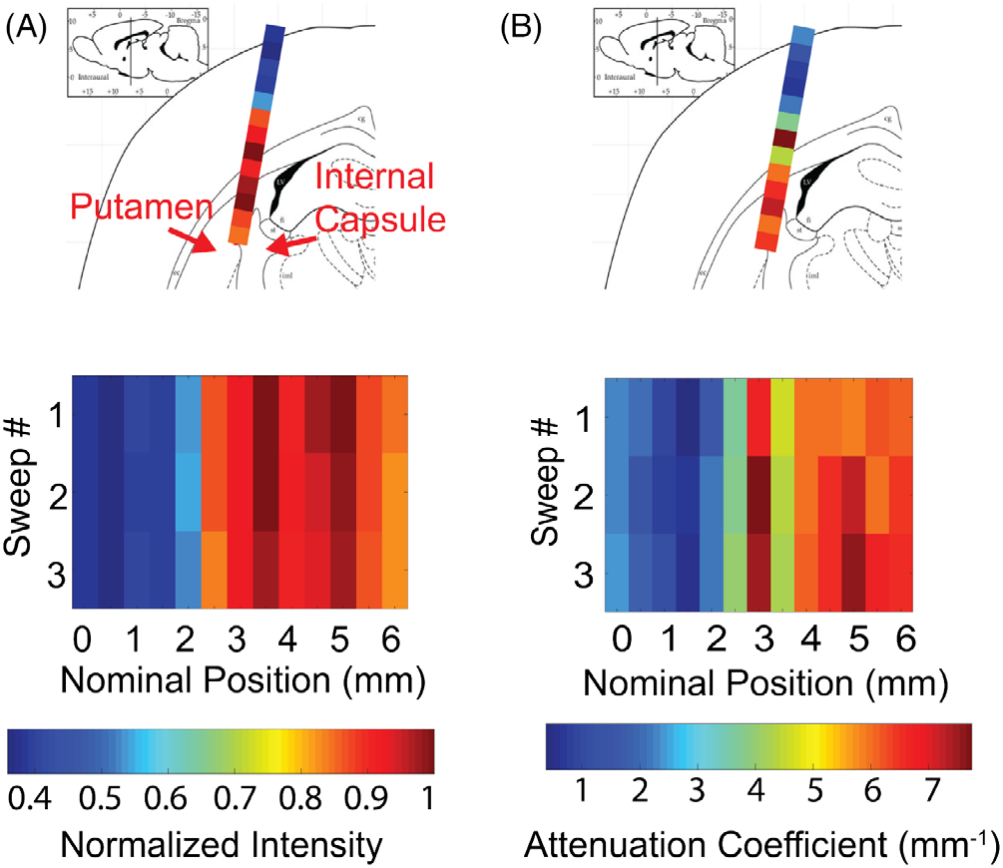
(A) Average intensity and (B) attenuation coefficient of the optical coherence tomography (OCT) scan along the insertion tract. The tract goes through cortex, corpus callosum and into striatum. The order of the structures appearing matches with brightness and attenuation coefficient appearing in OCT scan. Regions with dense myelinated fiber have bright intensity and high attenuation and regions like cortex have the weakest for both metrics. Results from three repeated sweeps are presented to validate the data consistency

Figure 10 shows the average intensity and the attenuation coefficient of the OCT scan along the tract. These properties correlate with the relative anatomic positions of the brain regions. For example, the weakest intensity and the lowest attenuation were at the shallowest penetration depths, corresponding to the location of the cortex, while the brightest intensity and the highest attenuation were closest to the corpus callosum along the tract. A bright intensity region appeared near 5 mm penetration, which corresponded to the interface between the putamen and internal capsule, a region with dense myelinated fibers. Such results were repeatable over three sequential scans at the same position. Compared to the ex vivo data, the in vivo data shows a lower attenuation for the cortex region and corpus callosum (1‐4 vs 4‐8 mm^−1^). This is expected as the attenuation of certain brain regions has been reported to increase after exposing to room temperature saline 56.

**Figure 11:**
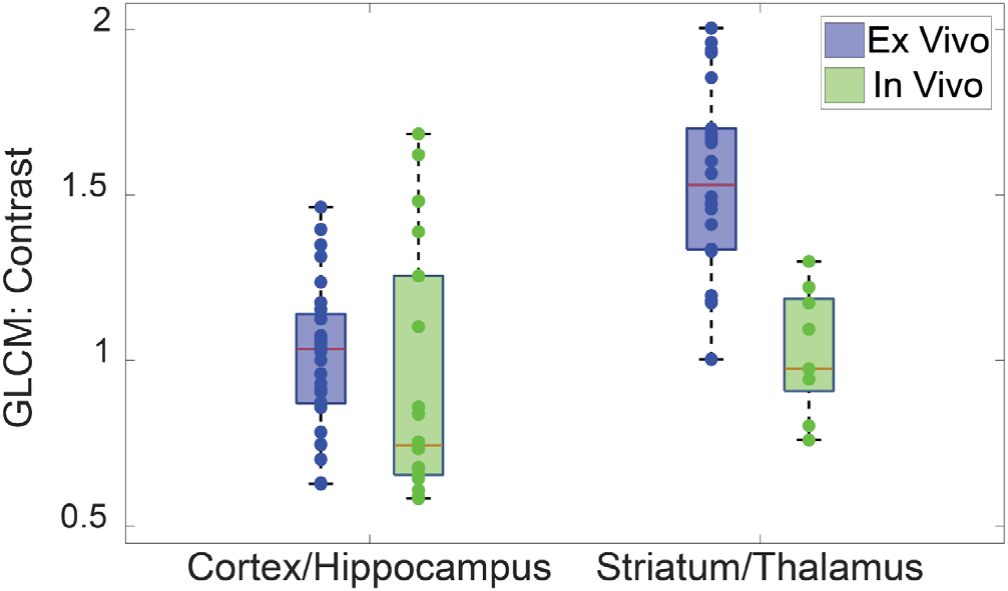
Comparison of the Gray Level Co‐Occurrence Matrix (GLCM) contrast calculated from ex vivo and in vivo data. in vivo data have lower contrast for both cortex and thalamus/striatum. Contrast values for in vivo data are extracted from the insertion tract presented in the article

Figure 11 shows that in vivo data generally have lower GLCM contrast than the ex vivo data. Also, the standard deviation of contrast value for in vivo cortex is 50% of the mean. The high variability makes it difficult to directly compare in vivo with ex vivo data. These results can be explained by motion artifact from natural biological processes such as cerebrospinal fluid (CSF) pulsation and respiratory motion of the rat. The experiments in 57 showed that transverse and axial motion can reduce the image resolution which leads to a reduced contrast. Also, axial motion increases the depth error and decreases the SNR of the image, which can distort texture information. Since the acquisition time was 8 seconds, motion artifact would reduce the image quality. The drift in the attenuation factor and the GLCM properties posed a challenge in transferring the classification algorithm developed in the previous section to in vivo data.

## 4 DISCUSSION

We have presented the first demonstration of FF‐SS‐OCT with >1 μm wavelength light as well as the first demonstration of such a system with an endoscopic probe which can be inserted several millimeters into tissue. With a mm‐range penetration depth, a 400 μm DOF, 6.5 μm transverse and 14 μm axial resolutions, the OCT system presented offers imaging capabilities and a form factor that are unique compared to other imaging modalities and match the requirements for DBS procedures. In comparison with the work presented by Liang et al 32, this system was able to generate 3D images of the tissue with higher transverse resolution, which also provided additional details and features to aid in the classification of different brain regions. A framework for analyzing the obtained scans was also developed, including a classification algorithm for identifying different types of brain tissue for ex vivo samples.

Although individual neurons were not imaged, the system captured macro‐features that were related to the tissue composition of the brain regions. The repeatability measure applied to frozen ex vivo samples demonstrated that these macro‐features were repeatable over sequential scan with the system. Moreover, the intensity‐ and texture‐based features extracted from images in fresh ex vivo samples were consistent across tissue samples which led to a classification test accuracy of 75%. Potential sources of error are the variability between tissues (ie, density and orientation of myelinated axon) and the change of optical properties of brain regions over time in ex vivo samples. As reported previously 56, the attenuation coefficient of some regions of the brain, like cortex, can increase by two times within 30 minutes when only exposed to saline.

For the in vivo results, 2D OCT can be used in the future to validate if the dark shadows (eg, in Figure 9B,C) are blood vessels by measuring the intensity variance 58. The observed average intensity and the attenuation coefficient of the in vivo data exhibited correlation with the structures along the tract, which is promising for using the FF‐SS‐OCT system with the developed algorithm to classify brain regions in live brain imaging. However, directly applying the same classifier developed for ex vivo samples to in vivo samples can lead to inaccurate results. The main limiting factor for in vivo imaging was the degradation of the image quality due to vibrations from natural biological processes (eg, CSF pulsation and blood flow). This is evidenced by the reduced contrast of the in vivo data set compared to the ex vivo data set. One way to address this issue would be to acquire scans faster by using cameras with higher frame rates and lasers with a higher wavelength sweep speed.

Several other improvements can be made to generalize ex vivo classification model for in vivo data. First, acquiring ex vivo data in an air‐cooled environment at 2° to 4° would help to maintain the consistency of optical properties between ex vivo and in vivo samples 56. Second, a more complex classification model using deep learning methods would lead to more robust classification. Incorporating information like the penetration depth or preoperative MRI images can also counter inaccurate classification due to tissue variability, such as the fiber tract orientation with respect to the endoscope. Lastly, having a metal casing with a sharp tip would prevent tissue residue from being stuck on the endoscope.

Finally, for clinical settings, the optical system must be miniaturized and packaged with a longer endoscope similar to the prototype developed by Benoit a la Guillaume et al 46, but with longer imaging depth. Despite the limitations of our system and measurements, to our knowledge, our findings currently have no direct counterpart in the literature, and can be refined further for greater fidelity.

## 5 CONCLUSION

This work introduced the first full‐field SS‐OCT system with an endoscopic probe, operating in the wavelength range centered at 1310 nm. The achieved transverse and axial resolution is comparable with some of the previously demonstrated systems 32, 42, 59, and it presents additional advantages over other higher‐resolution systems 28, 45, 46, in particular due to the relatively thin endoscopic tip and the long imaging depth. The system has the advantage of using components developed for telecommunications. The proof‐of‐concept system enabled images and tomographies of rodent brains to be acquired with a transverse resolution of 6.5 μm and axial resolution of 14 μm in tissue. Applying classification on attenuation coefficient and GLCM features of the OCT images, regions of the brain, that is, cortex, corpus callosum and striatum/thalamus, can be accurately identified in ex vivo samples, which may translate to clinical applications such as guidance in keyhole tumor biopsy and DBS electrode implantation. For future work, the system can be miniaturized using original equipment manufacturer components and compacted into a slim, handheld form factor, similarly to the ones recently demonstrated for other OCT systems 46, 60. Additional work on upgrading the acquisition speed of the OCT and better methodology to obtain high quality data would improve the accuracy and extend the system to in vivo measurements.

## CONFLICT OF INTEREST

The authors declare that there are no conflicts of interest related to this article.

## Supporting information


**Appendix S1:** Supporting information

## References

[1] E. B. Claus , Clin. Oncol. 2012, 9(2), 79. 10.1038/nrclinonc.2011.179 22143137

[2] S. Noachtar , I. Borggraefe , Epilepsy Behav. 2009, 15, 66. 19236942 10.1016/j.yebeh.2009.02.028

[3] C. Andrews , I. Aviles‐Olmos , M. Hariz , T. Foltynie , J. Neurol. Neurosurg. Psychiatry 2010, 81(12), 1383. 20841370 10.1136/jnnp.2010.207993

[4] A. L. Benabid , Curr. Opin. Neurobiol. 2003, 13(6), 696. 14662371 10.1016/j.conb.2003.11.001

[5] J. S. Perlmutter , J. W. Mink , Annu. Rev. Neurosci. 2006, 29, 229. 16776585 10.1146/annurev.neuro.29.051605.112824 PMC4518728

[6] F. M. Skidmore , R. L. Rodriguez , H. H. Fernandez , W. K. Goodman , K. D. Foote , M. S. Okun , CNS Spectr. 2006, 11(7), 521. 16816792 10.1017/s1092852900013559

[7] K. J. Burchiel , S. McCartney , A. Lee , A. M. Raslan , J. Neurosurg. 2013, 119(2), 301. 23724986 10.3171/2013.4.JNS122324

[8] Z. Cui , L. Pan , H. Song , X. Xu , B. Xu , X. Yu , Z. Ling , J. Neurosurg. 2015, 124, 1. 26274983 10.3171/2015.1.JNS141534

[9] L. Thomas Foltynie , I. Zrinzo , E. Martinez‐Torres , E. Tripoliti , E. Petersen , I. Holl , M. Aviles‐Olmos , M. Jahanshahi , P. L. Hariz , J. Neurol. Neurosurg. Psychiatry 2011, 82(4), 358. 20571041 10.1136/jnnp.2010.205542

[10] R. M. Richardson , J. L. Ostrem , P. A. Starr , Stereotact. Funct. Neurosurg. 2009, 87(5), 297. 19641340 10.1159/000230692

[11] E. D. Flora , C. L. Perera , A. L. Cameron , G. J. Maddern , Mov. Disord. 2010, 25, 1550. 20623768 10.1002/mds.23195

[12] R. Verhagen , L. J. Bour , V. J. J. Odekerken , P. van den Munckhof , P. Richard Schuurman , R. de Bie , Brain Sci. 2019, 9(3), 51. 10.3390/brainsci9030051 PMC6469020 30832214

[13] J. Jimenez‐Shahed , M. York , E. M. Smith‐Gloyd , J. Jankovic , A. Viswanathan , Mov. Disord. 2014, 29, S1.

[14] A. Gorgulho , A. A. F. De Salles , L. Frighetto , E. Behnke , J. Neurosurg. 2005, 102(5), 888. 15926715 10.3171/jns.2005.102.5.0888

[15] T. M. Kinfe , J. Vesper , Acta Neurochir. Suppl. 2013, 117, 27. 23652653 10.1007/978-3-7091-1482-7_5

[16] W. Drexler , J. G. Fujimoto , Prog. Retin. Eye Res. 2008, 27, 45. 18036865 10.1016/j.preteyeres.2007.07.005

[17] T. Xie , G. Liu , K. Kreuter , S. Mahon , H. Colt , D. Mukai , G. M. Peavy , Z. Chen , M. Brenner , J. Biomed. Opt. 2009, 14(6), 064045. 20059283 10.1117/1.3275478 PMC2809499

[18] T. N. Ford , G. M. Solomon , H. M. Leung , G. J. Tearney , D. Cui , L. Liu , S. E. Birket , S. M. Rowe , C. Hyun , B. Yin , K. K. Chu , J. A. Gardecki , Opt. Lett. 2017, 42, 867. 28198885 10.1364/OL.42.000867 PMC5665567

[19] K. Park , N. H. Cho , J. H. Jang , S. H. Lee , P. Kim , M. Jeon , S. A. Boppart , J. Kim , W. Jung , Appl. Opt. 2017, 56, D115. 28375378 10.1364/AO.56.00D115 PMC5508522

[20] O. Thouvenin , C. Apelian , A. Nahas , M. Fink , C. Boccara , Appl. Sci. 2017, 7(3), 236.

[21] X. Fu , D. Patel , H. Zhu , G. MacLennan , Y. T. Wang , M. W. Jenkins , A. M. Rollins , Biomed. Opt. Express 2015, 6(4), 1164. 25909002 10.1364/BOE.6.001164 PMC4399657

[22] M. E. Pawlowski , S. Shrestha , J. Park , B. E. Applegate , J. S. Oghalai , T. S. Tkaczyk , Biomed. Opt. Express 2015, 6(6), 2246. 26114043 10.1364/BOE.6.002246 PMC4473758

[23] J. S. Iyer , S. A. Batts , K. K. Chu , M. I. Sahin , H. M. Leung , G. J. Tearney , K. M. Stankovic , Sci. Rep. 2016, 6, 33288. 27633610 10.1038/srep33288 PMC5025881

[24] S. G. Proskurin , S. V. Frolov , Biomed. Eng. 2012, 46(3), 96.

[25] I.‐K. Jang , B. E. Bouma , D.‐H. Kang , S.‐J. Park , S.‐W. Park , K.‐B. Seung , K.‐B. Choi , M. Shishkov , K. Schlendorf , E. Pomerantsev , S. L. Houser , H. T. Aretz , G. J. Tearney , J. Am. Coll. Cardiol. 2002, 39(4), 604. 11849858 10.1016/s0735-1097(01)01799-5

[26] M. S. Jafri , S. Farhang , R. S. Tang , N. Desai , P. S. Fishman , R. G. Rohwer , C.‐M. Tang , J. M. Schmitt , J. Biomed. Opt. 2005, 10(5), 051603. 16292951 10.1117/1.2116967

[27] J. B. Arous , J. Binding , J.‐F. Leger , M. Casado , P. Topilko , L. Bourdieu , S. Gigan , A. C. Boccara , J. Biomed. Opt. 2011, 16(11), 116012. 22112117 10.1117/1.3650770

[28] O. Assayag , K. Grieve , B. Devaux , F. Harms , J. Pallud , F. Chretien , C. Boccara , P. Varlet , NeuroImage Clin. 2013, 2, 549. 24179806 10.1016/j.nicl.2013.04.005 PMC3778248

[29] S. W. Jeon , M. A. Shure , K. B. Baker , D. Huang , A. M. Rollins , A. Chahlavi , A. R. Rezai , J. Neurosci. Methods 2006, 154(1‐2), 96. 16480773 10.1016/j.jneumeth.2005.12.008 PMC1769312

[30] V. J. Srinivasan , H. Radhakrishnan , J. Y. Jiang , S. Barry , A. E. Cable , Opt. Express 2012, 20(3), 2220. 22330462 10.1364/OE.20.002220 PMC3306182

[31] V. J. Srinivasan , J. Y. Jiang , M. A. Yaseen , H. Radhakrishnan , W. Wu , S. Barry , A. E. Cable , D. A. Boas , Opt. Lett. 2010, 35(1), 43. 20664667 10.1364/OL.35.000043 PMC2912612

[32] C.‐P. Liang , J. Wierwille , T. Moreira , G. Schwartzbauer , M. S. Jafri , C.‐M. Tang , Y. Chen , Opt. Express 2011, 19(27), 26283. 22274213 10.1364/OE.19.026283 PMC3297117

[33] M. A. Choma , M. V. Sarunic , C. Yang , J. Izatt , Opt. Express 2003, 11(18), 2183. 19466106 10.1364/oe.11.002183

[34] M. Harfouche , N. Satyan , G. Rakuljic , A. Yariv , J. Opt. Soc. Am. 2015, 3, 3.

[35] M. V. Sarunic , S. Weinberg , J. A. Izatt , Opt. Lett. 2006, 31, 1462. 16642139 10.1364/ol.31.001462

[36] T. Bonin , P. Koch , G. Hüttmann , G. Hüttmann , Optical Coherence Tomography and Coherence Techniques V, OSA, Washington, DC 2011, p. 80911K.

[37] L. A. Sordillo , Y. Pu , S. Pratavieira , Y. Budansky , R. R. Alfano , J. Biomed. Opt. 2014, 19(5), 056004. 24805808 10.1117/1.JBO.19.5.056004

[38] W.‐F. Cheong , S. A. Prahl , A. J. Welch , IEEE J. Quantum Electron. 1990, 26(12), 2166.

[39] C. L. Tsai , J. C. Chen , W. J. Wang , J. Med. Biol. Eng. 2001, 21(1), 7.

[40] S. L. Jacques , Phys. Med. Biol. 2013, 58(11), 5007. 10.1088/0031-9155/58/11/R37 23666068

[41] K. Bizheva , A. Unterhuber , B. Hermann , B. Považay , H. Sattmann , A. F. Fercher , W. Drexler , M. Preusser , H. Budka , A. Stingl , T. Le , J. Biomed. Opt. 2005, 10(1), 011006. 10.1117/1.1851513 15847572

[42] H. J. Böhringer , E. Lankenau , F. Stellmacher , E. Reusche , G. Hüttmann , A. Giese , Acta Neurochir. 2009, 151(5), 507. 19343270 10.1007/s00701-009-0248-y PMC3085760

[43] H. J. Böhringer , D. Boller , J. Leppert , U. Knopp , E. Lankenau , E. Reusche , G. Hüttmann , A. Giese , Lasers Surg. Med. 2006, 38(6), 588. 16736504 10.1002/lsm.20353

[44] S. A. Boppart , S. A. Boppart , M. E. Brezinski , C. Pitris , C. Pitris , J. G. Fujimoto , Neurosurgery 1998, 43(4), 834. 9766311 10.1097/00006123-199810000-00068

[45] C. Magnain , J. C. Augustinack , E. Konukoglu , M. P. Frosch , S. Sakadžic , A. Varjabedian , N. Garcia , V. J. Wedeen , D. A. Boas , B. Fischl , Neurophotonics 2015, 2(1), 015004. 25741528 10.1117/1.NPh.2.1.015004 PMC4346095

[46] E. Benoit a la Guillaume , F. Martins , C. Boccara , F. Harms , J. Biomed. Opt. 2016, 21(2), 026005. 10.1117/1.JBO.21.2.026005 26857471

[47] C. R. Butson , C. C. McIntyre , J. Neural Eng. 2005, 3(1), 1. 16510937 10.1088/1741-2560/3/1/001 PMC2583360

[48] J. A. Izatt , M. A. Choma , A.‐H. Dhalla , Optical Coherence Tomography: Technology and Applications, Berlin, Heidelberg: Springer, 2015, p. 65.

[49] G. Paxinos , C. Watson , The Rat Brain in Stereotaxic Coordinates, Vol. 547612, Amsterdam, Netherlands: Academic Press, 2006, p. 170.

[50] Z. Wang , A. C. Bovik , H. R. Sheikh , E. P. Simoncelli , et al., IEEE Trans. Image Process. 2004, 13(4), 600. 15376593 10.1109/tip.2003.819861

[51] R. M. Haralick , I.'h. Dinstein , K. Shanmugam , IEEE Trans. Syst. Man Cybern. 1973, SMC‐3(6), 610.

[52] H. Wang , C. Magnain , S. Sakadžić , B. Fischl , D. A. Boas , Biomed. Opt. Express 2017, 8(12), 5617. 29296492 10.1364/BOE.8.005617 PMC5745107

[53] Y. Xie , L.‐A. Harsan , T. Bienert , R. D. Kirch , D. Von Elverfeldt , U. G. Hofmann , Biomed. Opt. Express 2017, 8(2), 593. 28270970 10.1364/BOE.8.000593 PMC5330575

[54] S. Mika , G. Ratsch , J. Weston , B. Scholkopf , K.‐R. Mullers , in *Neural Networks for Signal Processing IX: Proc. of the 1999 IEEE Signal Processing Society Workshop (cat. no. 98th8468)*, IEEE, 1999, pp. 41–48.

[55] S. Palfi , J. M. Gurruchaga , G. S. Ralph , H. Lepetit , S. Lavisse , P. C. Buttery , C. Watts , J. Miskin , M. Kelleher , S. Deeley , H. Iwamuro , J. P. Lefaucheur , C. Thiriez , G. Fenelon , C. Lucas , P. Brugières , I. Gabriel , K. Abhay , X. Drouot , N. Tani , A. Kas , B. Ghaleh , P. le Corvoisier , P. Dolphin , D. P. Breen , S. Mason , N. V. Guzman , N. D. Mazarakis , P. A. Radcliffe , R. Harrop , S. M. Kingsman , O. Rascol , S. Naylor , R. A. Barker , P. Hantraye , P. Remy , P. Cesaro , K. A. Mitrophanous , Lancet 2014, 383(9923), 1138. 24412048 10.1016/S0140-6736(13)61939-X

[56] E. B. Kiseleva , Y. V. Korzhimanova , A. A. Moiseev , K. S. Yashin , L. B. Timofeeva , G. V. Gelikonov , E. V. Zagaynova , N. D. Gladkova , Laser Phys. Lett. 2019, 16(4), 045602.

[57] S. H. Yun , G. J. Tearney , J. F. De Boer , B. E. Bouma , Opt. Express 2004, 12(13), 2977. 19483816 10.1364/opex.12.002977 PMC2752339

[58] V. J. Srinivasan , S. Sakadžić , I. Gorczynska , S. Ruvinskaya , W. Weicheng , J. G. Fujimoto , D. A. Boas , Opt. Express 2010, 18(3), 24774. 10.1364/OE.18.002477 PMC2837842 20174075

[59] T. Bonin , G. Franke , M. Hagen‐Eggert , P. Koch , G. Hüttmann , Opt. Lett. 2010, 35, 3432. 20967090 10.1364/OL.35.003432

[60] S. Kim , M. Crose , W. J. Eldridge , B. Cox , W. J. Brown , A. Wax , Biomed. Opt. Express 2018, 9(3), 1232. 29541516 10.1364/BOE.9.001232 PMC5846526

