# Supplementary material for: Full‐field swept‐source optical coherence tomography and neural tissue classification for deep brain imaging
Source: J Biophotonics. 2019 Dec 2;13(2):e201960083. doi: 10.1002/jbio.201960083 (PMC7065632; doi:10.1002/jbio.201960083)
Supplement: Supplementary file 1 — Appendix S1: Supporting information [file JBIO-13-e201960083-s001.docx]

**Supporting information for “Full-field swept-source optical coherence tomography and neural tissue classification for deep brain imaging”**

ILAN FELTS ALMOG†1,2 | FU DER CHEN†1,2 | SUHAN SENOVA2,3,4 | ANTON FOMENKO2 | ELISE GONDARD2 | WESLEY D. SACHER1,5 | ANDRES M. LOZANO2,6 | JOYCE K. S. POON1,2,5

^1^­Edward S. Rogers Sr. Department of Electrical and Computer Engineering, University of Toronto, Toronto, Ontario, Canada

2Krembil Research Institute, Toronto Western Hospital, Toronto, Ontario, Canada

3Department of Neurosurgery, Centre Hospitalier Universitaire Henri-Mondor, APHP, Créteil, France

4INSERM Unit 955, Institut Mondor de Recherche Biomédicale, Université Paris-Est, Créteil, France

5Max Planck Institute of Microstructure Physics, Halle, Germany

6Division of Neurosurgery, Department of Surgery, Toronto Western Hospital,Toronto, Ontario, Canada

† These authors contributed equally to the work.

*Email: FC: [fuder.chen@mail.utoron to.ca](mailto:fuder.chen@mail.utoron%20to.ca)

**S1. System characterization**


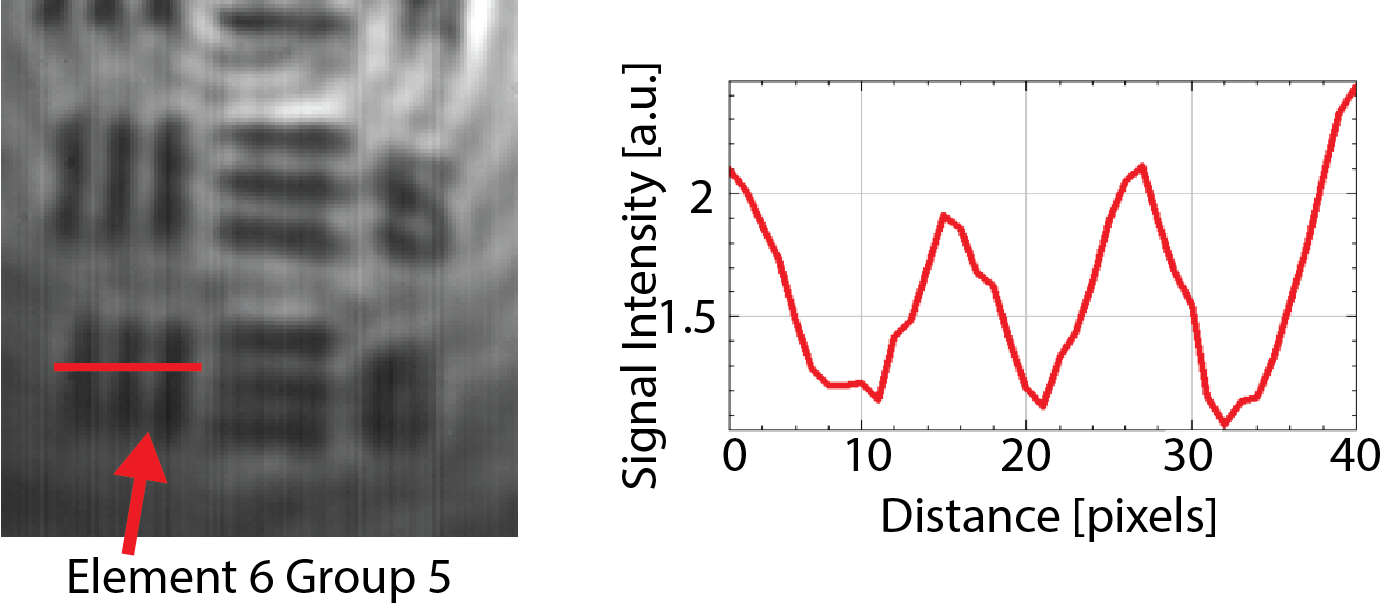


**Figure S1.1.** Image of the smallest resolvable features from a USAF-1951 resolution target illuminated with a broad band NIR source.

USAF-1951 resolution target was used to verify the transverse resolution of the proposed system. The sample was illuminated with a broadband near-infrared (NIR) source to avoid interference pattern affecting the measurement. Figure S1.1 shows the image of Element 5-6 (line separation: 8.77 μm) captured with the OCT system. 10-20% contrast is presented by the intensity line profile across the red line. This result confirms the system has a transverse resolution of at least 8.77 μm in air.


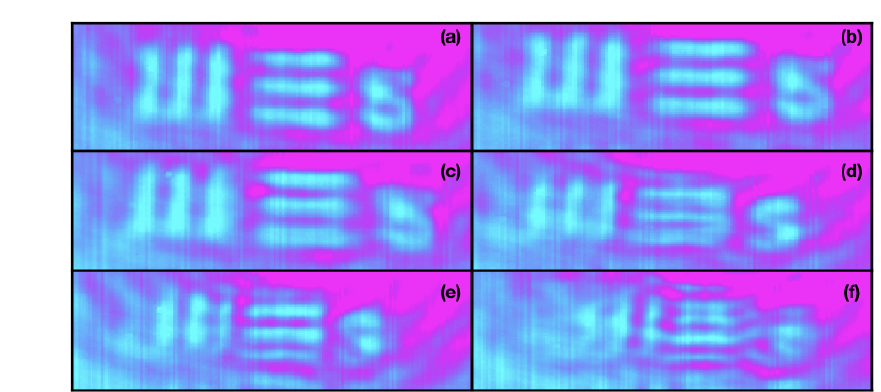


**Figure S1.2.** Images of Element 5-5 on a USAF-1951 resolution target positioned at: (a) best focus, (b) 50 μm away, (c)150 μm away, (d) 250 μm away, (e) 300 μm away and (f) 400 μm away. The vertical line merges in between 150um and 250um away from the initial position.

Next, we used the resolution target to measure the depth of field (DOF) of the system. Element 5-5 was initially positioned at the best focus. Then, we image the same element at different positions away from the initial point. From Figure S1.2, we determined 200 μm to be half DOF since the vertical line merges in between 150 μm away and 250 μm away from the initial position. Similar blur distance is expected along the opposite direction; thus, the DOF of the system is determined to be 400 μm.


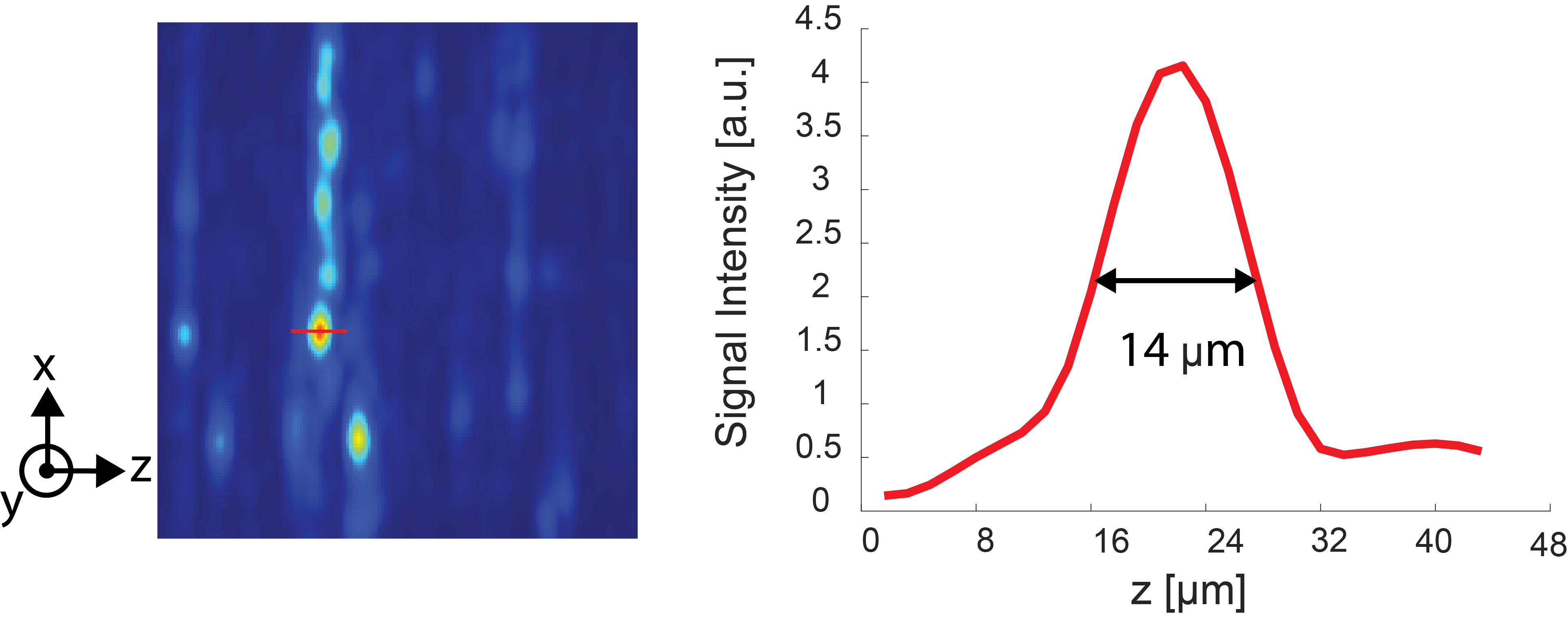


**Figure S1.3.** Depth profile of an OCT-resolved bead showing a full-width-at-half-maximum (FWHM) of 14 μm, with the refractive index n of the agarose block to be 1.34.

Last, we tested the system with the OCT-resolved scanning of the phantom with suspended beads with diameter of 55±1 μm. Figure S1.3 shows the axial profile of the bead. We assume a single reflection from a limited portion of the incident surface of the high-refractive-index beads. The axial resolution determined from the FWHM of the depth profile in Figure S1.3 is 14 μm in tissue.

**S2. Repeatability of tissue structures beyond DOF of the system**

**
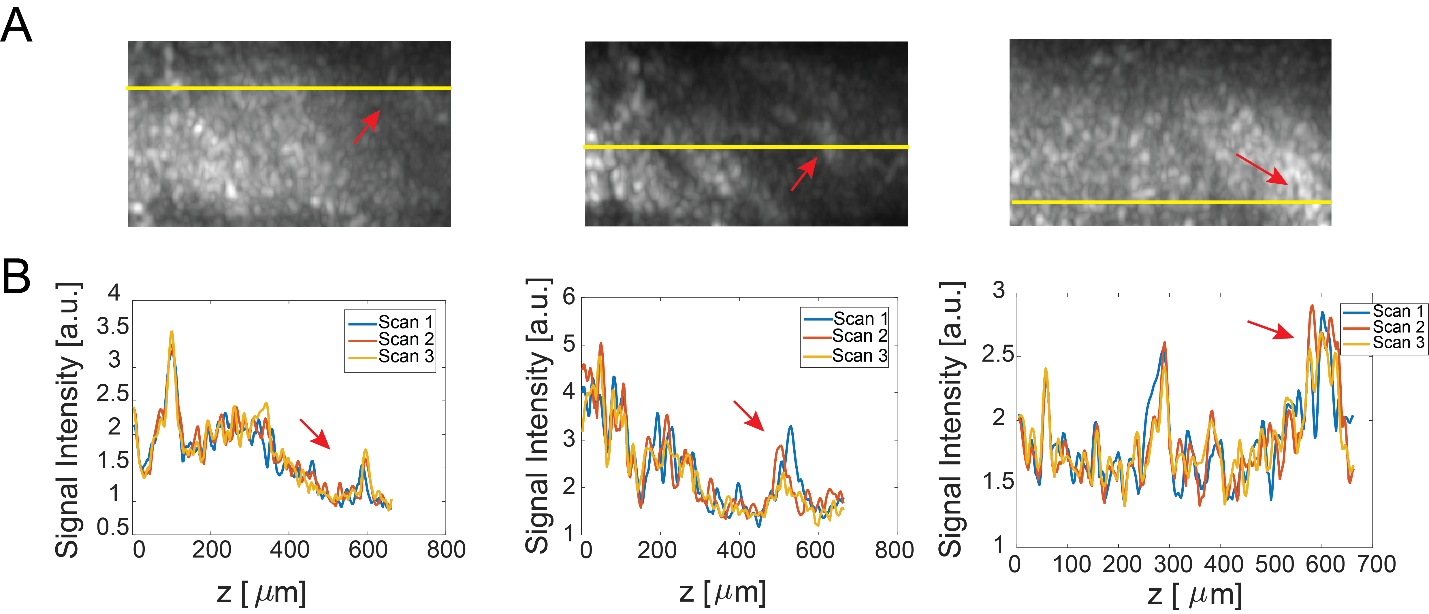
**

**Figure S2.1.** a) Images of maximum projection image of ex vivo striatum with some distinct structures, labeled by red arrows, positioned deeper than the DOF of the system. b) Line profile across the yellow for 3 repeated scans at the same position, confirming the repeatability of the structures.

Although we claim the DOF of the OCT system to be 400 μm, the system can still capture some structures deeper than DOF in heterogeneous brain regions like striatum. Three ex vivo striatum samples are presented in Figure S2.1 to demonstrate the case. The structures labeled by the red arrow are repeatable over 3 sequential scans as shown by the intensity profile across the yellow line. The maximum depth of the image is extended to 700 μm to preserve these features.
